# The effect of the number of endometrial CD138+ cells on the pregnancy outcomes of infertile patients in the proliferative phase

**DOI:** 10.3389/fendo.2024.1437781

**Published:** 2025-01-22

**Authors:** Yuye Li, Shuyi Yu, Wenjuan Liu, Yawen Chen, Xiaobing Yang, Juanhua Wu, Mingjuan Xu, Guanying You, Ruochun Lian, Chunyu Huang, Wanru Chen, Yong Zeng, Fenghua Liu, Lianghui Diao

**Affiliations:** ^1^ Shenzhen Key Laboratory of Reproductive Immunology for Peri-implantation, Shenzhen Zhongshan Institute for Reproductive Medicine and Genetics, Shenzhen Zhongshan Obstetrics and Gynecology Hospital (formerly Shenzhen Zhongshan Urology Hospital), Shenzhen, China; ^2^ Guangdong Engineering Technology Research Center of Reproductive Immunology for Peri-implantation, Shenzhen, China; ^3^ Department of Reproductive Medical Center, Guangdong Women and Children Hospital, Guangzhou, Guangdong, China

**Keywords:** infertile women, IVF-ET, CE, CD138, pregnancy outcome, proliferative phase, midluteal phases

## Abstract

**Objective:**

This study was conducted to determine the influence of the number of CD138^+^ cells in the proliferative endometrium on pregnancy outcomes.

**Methods:**

This retrospective cohort study was conducted from January to August 2018. A total of 664 infertile women who were not diagnosed with chronic endometritis (CE) and who had not received the respective antibiotic treatment were studied. Immunostaining was performed for the plasmacyte marker CD138. The number of CD138+ cells was compared in the proliferative and mid-luteal phases of the same patients without antibiotic therapy. Infertile patients were separated into three groups based on the number of positive lesions [the number of high power fields (HPFs) containing no less than five CD138^+^ cells]: 0 (n = 474), 1-2 (n = 125), and **≥**3 positive lesions (n = 104). The pregnancy outcomes of the infertile women undergoing *in vitro* fertilization-embryo transfer (IVF-ET) among the three groups were then compared.

**Results:**

There was a much higher level of CD138+ cells during proliferation than during the mid-luteal phase (P <0.0001). Pregnancy outcomes did not differ between the groups with 0 and 1-2 positive lesions. However, the ≥3 positive lesions group (*P* =0.006, *P* =0.029) had significantly lower ongoing pregnancy and live birth rates compared with the no positive lesion group. Although the 0 and ≥3 positive lesions groups showed a trend toward higher rates of clinical pregnancy (*P* =0.132), these differences failed to reach statistical significance. After age, body mass index (BMI), and clinical features were adjusted for, the *≥*3 positive lesions group showed significantly lower live birth rates (aOR, 1.84; 95%CI, 1.08-3.15; *P* =0.026), clinical pregnancy (adjusted odds ratio (aOR), 1.78; 95% CI, 1.06-2.95; *P* =0.028), and ongoing pregnancy (aOR, 1.85; 95% CI, 1.09-3.15; *P* =0.024). The analysis demonstrated that the smallest number of stromal CD138^+^ cells suggestive of CE patients requiring treatment was defined as *≥* 3 positive lesions during the proliferation.

**Conclusions:**

Different diagnostic criteria for CE should be created for the proliferative and mid-luteal phases. The analysis demonstrated that the smallest number of stromal CD138^+^ cells suggestive of CE patients was defined as *≥* 3 positive lesions during the proliferative phase.

## Introduction

Infertility, defined as the failure to achieve a pregnancy after 12 months or more of regular unprotected sexual intercourse, is a global reproductive health issue. Despite the rapid development of *in-vitro* fertilization and embryo transfer (IVF-ET) technology in recent years, the clinical pregnancy rate remains approximately 40%. Even if high-quality embryos are transferred, some patients still cannot successfully conceive. Research has found that approximately two-thirds of implantation failures are due to abnormal endometrial receptivity (1, 2). Multiple clinical studies have shown that chronic endometritis (CE) has become an important cause of pregnancy failure (3). Our previous study also showed that CE can significantly reduce the clinical pregnancy rate and live birth rate of infertile patients (4).

CE refers to a disease of continuous endometrial inflammation (5, 6) which primarily results from an infection with *Streptococcus* spp., *Enterococcus faecalis.*, *Staphylococcus* spp. and other pathogenic microorganisms (7–9). It is normally clinically asymptomatic or shows non-specific symptoms, and only features the infiltration of numerous endometrial stromal plasma cells (10). CE has a prevalence of 2.8%-56.8% among infertile women (5, 11–13). Previous data revealed that 5.3% (14), 10.4% (45/433), and 10.5% (29/275) of infertile, recurrent miscarriage and repeated implantation failure patients reported CE, respectively, according to the results of immunohistochemical detection of the endometrial cluster of differentiation 138 (CD138) (syndecan-1) (15). Nevertheless, no definitive diagnostic criteria have been established for CE, although experts agree that the presence of multiple CD138^+^ cells is the most specific and sensitive finding.

CD138, a cell membrane proteoglycan, serves as a matrix receptor and is mainly expressed on the surface of mature plasma and epithelial cells (16). Recommended as an immunohistochemical tissue marker specific for plasmacytes in humans, it has been used clinically for evaluating and characterizing plasma cells (17, 18). The number of CD138^+^ cells varies through the menstrual cycle of normal women (19). Lai et al. (20) reported that the expression of CD138 was downregulated in the endometrium stroma after ovulation and remained low throughout the luteal phase. This is consistent with a previous report that normal endometria in the mid-luteal phase usually showed negative or few CD138 staining (16). Given the fluctuating amount of CD138^+^ cells during different stages of the menstrual cycle, the diagnostic criteria for CE based on the amount of CD138^+^ cells may need to be adjusted following the phase of endometrial sampling.

In a previous study, the authors examined the link between the number of endometrial stromal CD138^+^ cells and infertile women’s pregnancy outcomes to offer CE diagnostic criteria of clinical relevance during the mid-luteal phase (4). The expression of no less than five CD138 ^+^ cells in one high power field (HPF) was shown to possibly affect the pregnancy outcomes of infertile patients. Based on this finding, the smallest number of stromal plasma cells suggestive of clinically related CE was suggested to be five CD138^+^ cells in no less than one out of 30 chosen HPFs (4). However, whether the samples collected during the proliferative phase require more CD138^+^ plasma cells to affect pregnancy outcomes remains uncertain.

Therefore, whether the amount of CD138^+^ plasma cells in the endometrial stroma was different between the proliferative and mid-luteal phases was analyzed in the present study. Additionally, the number of CD138^+^ cells in the endometrium suggestive of clinically treatable CE in the proliferative phase was defined.

## Materials and methods

This retrospective study was approved by the Ethics Committee of Shenzhen Zhongshan Obstetrics and Gynecology Hospital (SZZSECHU-F-2025001). Each endometrial biopsy was gathered with the written informed consent of the individual participants who allowed their endometrial tissues to be scientifically studied.

### Study population

**Figure 1:**
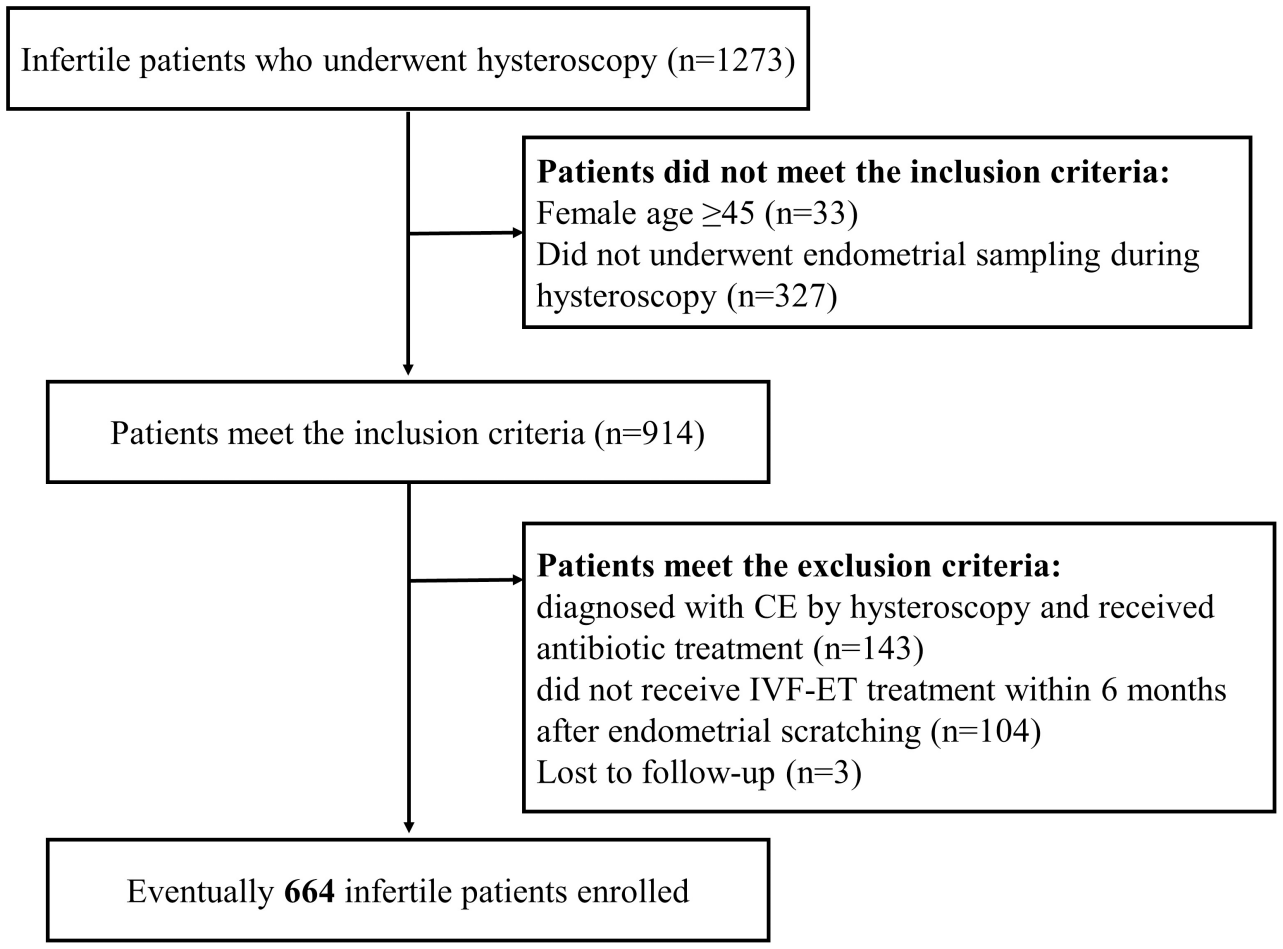
Flow chart of patient inclusion. A total of 1,273 infertile patients who underwent endometrial scraping between January and August 2018 were retrospectively analyzed in this study. Based on the inclusion and exclusion criteria, a total of 664 infertile patients were included in the study.

A retrospective analysis was conducted on infertile women who underwent IVF/intracytoplasmic sperm injection (ICSI) treatment at Shenzhen Zhongshan Obstetrics and Gynecology Hospital between January and August 2018. Infertile women who underwent IVF/ICSI treatment who had undergone a previous hysteroscopy were included in this study. The flowchart is presented in **Figure 1**. Infertile patients aged below 45 were included. The exclusion criteria were as follows: patients diagnosed with CE via hysteroscopy and receiving antibiotic treatment; patients who were not receiving IVF-ET treatment within 6 months after endometrial scratching or those without being followed up. Eventually, 664 infertile patients were enrolled. Each patient was included only once in the study.

**Figure 2:**
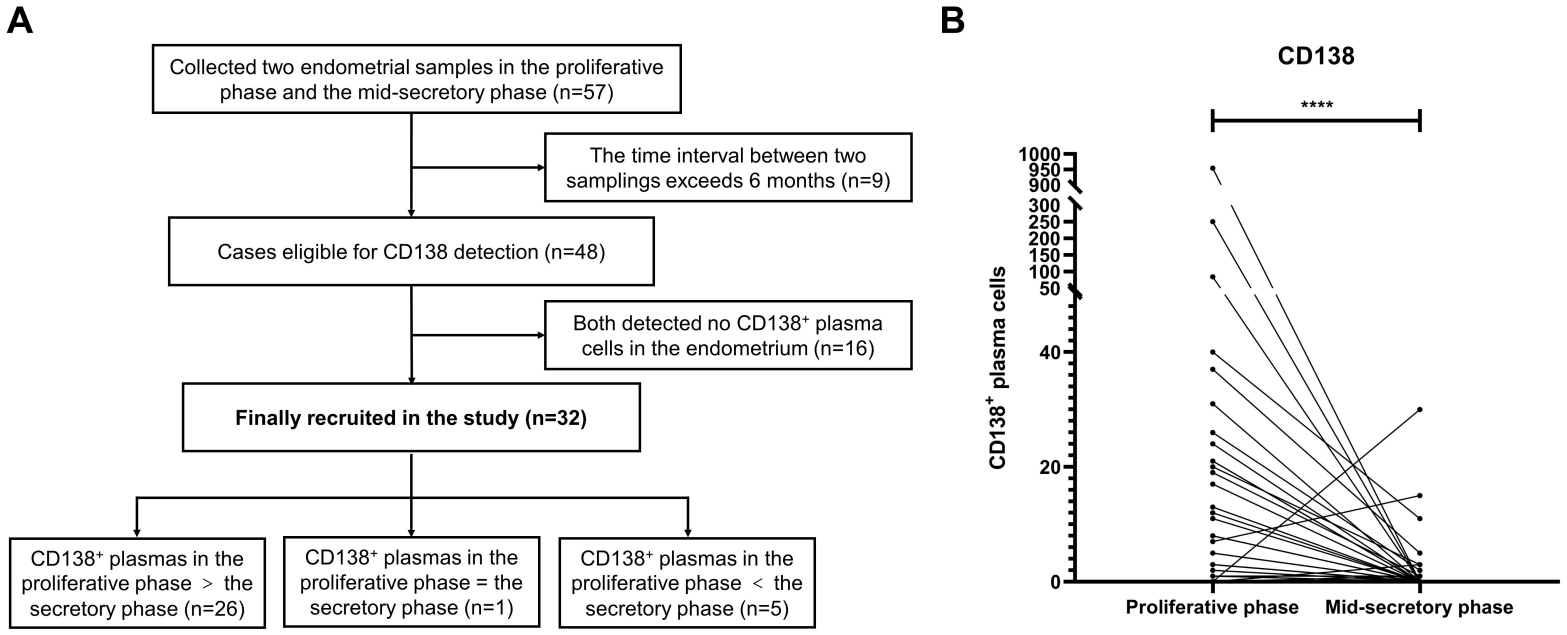
The number of CD138^+^ plasma cells in the proliferative and mid-luteal phase in the same patients without antibiotic therapy. **(A)** 57 infertile patients underwent hysteroscopy during the proliferative phase and endometrial scratching during the mid-luteal phase. **(B)** The number of CD138^+^ plasma cells in the proliferative and mid-luteal phase in the same patients ***P<0.001.

In addition, 57 enrolled patients participated in a clinical study that aimed at evaluating the effect of endometrial scratching during the mid-luteal phase on pregnancy outcomes. Their endometrial tissues were retained after obtaining informed consent. After the exclusion of patients with a time interval between two samplings exceeding 6 months and with antibiotic therapy detected from the proliferative and mid-luteal phases, 32 patients were recruited to compare the number of endometrial CD138^+^ cells in 30 HPFs between both phases (**Figure 2**).

### Endometrial biopsies

Hysteroscopy was performed on days 3-5 after menstruation for each participant in the preceding cycle before the prearranged treatment of IVF. CE by hysteroscopy was diagnosed by the presence of focal or diffuse endometrial hyperemia, stroma edema, and polyps with a size of less than 1 mm. Endometrial biopsies were taken by curette and then fixated for 4-6 hours in 10% neutral buffered formalin at ambient temperature. A paraffin embedding procedure was performed on all the endometrial tissues from the patients for subsequent research.

### Immunohistochemistry staining

The paraffin-embedded endometrial biopsy tissue was sliced into 4 µm sections, followed by deparaffinization in xylene and dehydration in alcohol. Immunohistochemistry (IHC) staining was conducted using a Leica Bond III automated immunostainer (Leica Biosystems, Welzlar, Germany) and Bond Polymer Refine Detection (DS9800, Leica Microsystems, Welzlar, Germany) following the protocol of the manufacturer. Briefly, the slides underwent 20 minutes of heating at 100°C in an ethylene diamine tetraacetic acid (EDTA) solution for the retrieval of antigens. The activity of endogenous peroxidases was blocked with 3% hydrogen peroxide (H_2_O_2_) in methanol for 10 minutes. Next, the sections underwent a 30-minute incubation with CD138 antibody at a dilution of 1:250 (Gene Tech, Shanghai, China). After that, the slides were rinsed and incubated for 30 mins with a secondary antibody conjugated with horseradish peroxidases. Immunostaining was performed by staining and counter-staining with 3, 3’-diaminobenzidine chromogen (DAB) and hematoxylin, respectively. Tissue-Tek Film Automated Coverslippers (Sakura Finetek, CA, USA) were utilized for cover-slipping the stained slides.

### Identification and count of CD138^+^ plasma cells

To diagnose CE, the sum of plasma cells was obtained by calculating the number of CD138^+^ cells in the endometrial stroma under an optical microscope (Nikon Microscope, Melville, New York) in 30 randomly chosen HPFs (200× magnification, 0.0625 mm^2^/fields).

Plasma cells were considered CD138^+^ cells when the cell membranes were strongly immunopositive, the cytoplasm demonstrated poor positive immunostaining, and the round nucleus was located on one side of cells, within which the condensed chromatin was organized in a radial way along the nuclear membrane to obtain a wheel pattern.

CD138^+^ cells were identified and counted by two experienced pathologists independently. Any disagreement between them was solved via discussions or a group discussion with a senior pathologist.

### IVF-ET outcome measurement

The primary outcome was live birth rate after one cycle of FET. Live birth was defined as the delivery of at least one newborn after 24 weeks of gestation exhibiting any sign of life (twins were a single count). Secondary outcomes were biochemical pregnancy rate, clinical pregnancy rate, and early miscarriage rate. Medically, a biochemical pregnancy means that an identifiable pregnancy is absent in an ultrasound examination although a pregnancy test of urine or blood hCG is positive. Biochemical pregnancy refers to a level of hCG > 5 on two occasions within 15 days or greater after the injection of hCG. Clinical pregnancy meant that a gestational sac was present sonographically at 7 to 8 weeks of gestation. An early miscarriage, or a first trimester miscarriage, is one that happens in the first 12 weeks of pregnancy.

### Statistical analysis

Data were expressed as mean ± standard deviation (SD) (data following a normal distribution), medians with interquartile ranges (data not following a normal distribution), and frequencies (percentages) for categorical variables. Multiple comparisons were performed using one-way analysis of variance (ANOVA) or the Kruskal–Wallis test for continuous variables and the Chi-square test for categorical variables.

The prognostic importance of CD138^+^ cells for patients’ pregnancy outcomes was identified using multivariable logistic regression models. Odds ratios (ORs) and adjusted ORs (aORs), their respective 95% confidence intervals (95% CIs) for the positive rate of HCG, and clinical pregnancy and live birth rates, were computed by less than 3 and 3 or more positive lesions.

Statistical Package for the Social Sciences (SPSS) Statistics version 20.0 (SPSS Inc., Chicago, Illinois (IL), USA) was adopted to perform all statistical analyses. It was considered that the *P*-value of *≤* 0.05 showed statistical significance.

## Results

### The number of CD138^+^ plasma cells during the proliferative and mid-luteal phases in the same patients without antibiotic therapy

The endometria of 57 infertile enrolled patients [mean (SD) age: 34.4(4.3) years] who underwent hysteroscopy during the proliferative phase were also scratched during the mid-luteal phase. Among them, nine patients failed to meet the criteria and were excluded. The number of CD138^+^ cells in 30 HPFs during the proliferative and mid-luteal phases of 48 patients who did not receive antibiotic therapy was assessed. After the exclusion of 16 patients whose endometria had no CD138^+^ cells in both the proliferative and mid-luteal phases, there was a significantly higher level of CD138^+^ cells in the proliferative phase than in the mid-luteal phase (*P <*0.0001, **Figure 2B**). In detail, the number of CD138^+^ cells were higher in the proliferative phase than in the mid-luteal phase among 26 patients. However, one patient presented the same amount of CD138^+^ cells during both the proliferative and mid-luteal phases, and five showed a lower number of CD138^+^ cells in the proliferative phase than in the mid-luteal one (**Figure 2A**).

### Clinical characteristics of infertile patients

To determine the connection between the number of CD138^+^ cells during the proliferative phase and the pregnancy outcomes of infertile women, 664 infertile women were enrolled in this retrospective study from January to August 2018 (**Figure 1**). We conducted an analysis of the number of patients exhibiting varying numbers of positive lesions (**Supplementary Table 1**) and observed a significantly smaller proportion of patients with ≥3 positive lesions compared to those with 0 or 1-2 positive lesions. Therefore, we choose three as the threshold. Infertile patients were classified into three groups according to the distribution of stromal CD138^+^ cells and the number of positive lesions (the number of HPFs containing no less than five CD138^+^ cells): 0 (n=453), 1-2 (n=113), and **≥** 3 (n=98) positive lesions. **Table 1** describes the clinical features of the included participants from different groups.

**Table 1:** Baseline characteristics of the study participants.

Baseline characteristic	No positive lesion (n=453)	1-2 positive lesions (n=113)	≥3 positive lesions (n=98)	*P-*value
Woman’s age				
Median (IQR), y	34.0 (31.0, 37.0)	34.0 (31.0, 37.0)	35.0 (31.0, 38.0)	0.249
≥35 (%)	212 (46.8)	50 (44.2)	52 (53.1)	0.412
Woman’s BMI				
Median (IQR), Kg/m^2^	21.5 (19.9, 23.5)	21.5 (19.5, 23.5)	21.1 (19.6, 23.3)	0.541
Underweight (BMI<18.5, %)	53 (11.7)	16 (14.2)	11 (11.2)	0.939
Normal weight (18.5≤BMI<24, %)	304 (67.1)	72 (63.7)	67 (68.4)	
Overweight and obese (BMI≥24, %)	96 (21.2)	25 (22.1)	20 (20.4)	
Infertility duration, Median (IQR), y	3.0 (2.0, 5.0)	3.5 (1.5, 5.0)	3.0 (2.0, 6.5)	0.368
Type of infertility				
Primary (%)	216 (47.7)	45 (39.8)	38 (38.8)	0.130
Secondary (%)	237 (52.3)	68 (60.2)	60 (61.2)	
Endometrial polyps	73 (16.1)	21 (18.6)	12 (12.2)	0.450
Cause of infertility				
Anatomic factor (%)	35 (7.7)	13 (11.5)	11 (11.2)	0.306
Ovarian factor (%)	6 (1.3)	2 (1.8)	2 (2.0)	0.843
Endometriosis (%)	14 (3.1)	4 (3.5)	2 (2.0)	0.805
Hysteromyoma (%)	8 (1.8)	4 (3.5)	2 (2.0)	0.501
Endocrine factor (%)	46 (10.2)	7 (6.2)	9 (9.2)	0.432
Male factor (%)	66 (14.6)	13 (11.5)	21 (21.4)	0.116
Mix factor (%)	120 (26.5)	27 (23.9)	15 (15.3)	0.064
Unexplained (%)	158 (34.9)	43 (38.1)	36 (36.7)	0.798
AMH, Median (IQR), ng/mL	2.5 (1.3, 4.4)	2.9 (1.5, 4.8)	2.3 (1.1, 3.5)	0.141
No. of transferred embryos, Median (IQR)	2 (1,2)	1 (1,2)^a*^	2 (1,2)	0.036
Embryo quality				
Cycles with high-quality embryos (%)	361 (79.7)	83 (73.5)	76 (77.6)	0.348
Cycles without high-quality embryos (%)	92 (20.3)	30 (26.5)	22 (22.4)	
Embryo type				
Cleavage (%)	130 (28.7)	21 (18.6) ^a*^	34 (34.7)	0.026
Blastocyst (%)	323 (71.3)	92 (81.4)	64 (65.3)	
Embryo transfer				
Fresh (%)	16 (3.5)	6 (5.3)	2 (2.0)	0.438
Frozen (%)	437 (96.5)	107 (94.7)	96 (98.0)	
Fertilization methods				
IVF (%)	350 (77.3)	95 (84.1)	81 (82.7)	0.185
ICSI (%)	103 (22.7)	18 (15.9)	17 (17.3)	

BMI, body mass index; AMH, anti-Müllerian hormone; IVF, *in-vitro* fertilization; ICSI, intracytoplasmic sperm injection.

Values were presented as median with quartiles or percentage (%). P-values were determined using the Kruskal–Wallis test or Pearson’s chi-square test. ^a^Statistically significant difference between the no positive lesion group and the 1-2 positive lesions group. ^*^ *P*<0.05.

The number of transferred embryos and embryo type varied among these groups. Women with no positive lesions had a higher number of embryos transferred and less blastocysts transferred than those with 1-2 positive lesions.

### Reproductive failure caused by ≥3 positive lesions of infertile women in the proliferative phase

The relationship between the density of endometrial CD138^+^ cells during the proliferative phase and the pregnancy outcomes of infertile patients was assessed. As shown in **Table 2** and **Supplementary Figure 1**, the analysis indicated that pregnancy outcomes did not differ between the 0 and 1-2 positive lesions groups. Nevertheless, the ≥3 positive lesions group (31.96% vs.46.00% or 48.21%, *P* =0.027; 45.92% vs. 58.28% or 63.72%, *P* =0.026; 32.99% vs. 48.22% or 49.11%, *P* = 0.019) showed a marked decline in live birth, clinical pregnancy, and ongoing pregnancy rates compared with the 0 and 1-2 positive lesions groups. The rate of biochemical pregnancy and early miscarriage showed no significant difference between the 0 or 1-2 groups and the ≥ 3 positive lesions group.

**Table 2:** The pregnancy outcomes in different groups.

Pregnancy outcome	No positive lesion (n=453)	1-2 positive lesions (n=113)	≥3 positive lesions (n=98)	P-value
Live birth rate (%)	46.00% (207/450)	48.21% (54/112)	31.96% (31/97)^a*,b*^	0.027^*^
Biochemical pregnancy rate (%)	67.11% (304/453)	72.57% (82/113)	59.18% (58/98) ^b*^	0.118
Clinical pregnancy rate (%)	58.28% (264/453)	63.72% (72/113)	45.92% (45/98) ^a*,b**^	0.026^*^
Ectopic gestation (n)	3	1	1	
Ongoing pregnancy rate (%)	48.22% (217/450)	49.11% (55/112)	32.99% (32/97) ^a**,b*^	0.019^*^
Early miscarriage rate (%)	16.67% (44/264)	22.22% (16/72)	26.67% (12/45)	0.207

Values were presented as percentages (%). *P*-values were determined using Pearson’s chi-square tests. ^a^Statistically significant difference between the no positive lesion group and the ≥3 positive lesions group. ^b^Statistically significant difference between the 1-2 positive lesion group and the ≥3 positive lesions group. ^*^ *P*<0.05, ^**^ *P*<0.01.

Based on the above findings, it was assumed that ≥ 3 positive lesions might influence pregnancy outcomes. As a result, the patients were re-grouped into ≥3 (n = 566) and < 3 positive lesions (n = 98). Subsequently, the results of pregnancy outcomes were compared between the <3 and ≥3 positive lesions groups. The data shown in **Supplementary Table 2** suggested that live birth, clinical pregnancy, and ongoing pregnancy rates in the ≥3 positive lesions group tended to decrease significantly (31.96% vs. 46.44%*, P* =0.005; 45.92% vs. 59.36%, *P* =0.013; 32.99% vs. 48.40%, *P* =0.005).

### Logistic regression analysis of the effect of ≥3 CD138^+^ plasma cell-positive lesions in the proliferation phase on pregnancy outcomes

Whether ≥3 CD138^+^ plasma cell-positive lesions was an independent risk factor for pregnancy outcomes was further demonstrated using a multivariate logistic regression analysis. The analysis showed that having ≥3 positive lesions was negatively correlated with pregnancy outcomes, as displayed in **Table 3**. Confounding factors such as the age and body mass index (BMI) of the woman, endometrial polyps, anti-Müllerian hormone (AMH), infertility duration and type, embryo characteristics (the number of transferred embryos, embryo quality, embryo type, and embryo transfer), and fertilization methods were adjusted for. After the adjustment for the age and BMI of the woman, the ≥3 positive lesions group exhibited a dramatic decline in live birth rates (aOR, 1.838; 95% CI, 1.074-3.145; *P* =0.026), clinical pregnancy (aOR, 1.771; 95% CI, 1.063–2.950; *P* = 0.028), and ongoing pregnancy (aOR 1.849; 95% CI, 1.086-3.150; *P* =0.024) compared with the <3 positive lesions group. However, the difference in the rate of biochemical pregnancy was not significant. In summary, the findings revealed that having ≥3 positive lesions was an independent risk factor influencing infertile patients’ pregnancy outcomes.

**Table 3:** Logistic regression analysis of the effect of CE on pregnancy outcomes.

Outcome	OR (95%CI)	*P-*value	aOR (95%CI)	*P-*value
Live birth	1.816 (1.149, 2.870)	0.011^*^	1.838 (1.074, 3.145)	0.026^*^
Biochemical pregnancy	1.434 (0.921, 2.232)	0.110	1.286 (0.759, 2.178)	0.350
Clinical pregnancy	1.681 (1.090, 2.591)	0.019^*^	1.771 (1.063, 2.950)	0.028^*^
Ongoing pregnancy	1.873 (1.189, 2.950)	0.007^**^	1.849 (1.086, 3.150)	0.024^*^

CE, chronic endometritis. OR, odds ratio. aOR, adjusted odds ratio. CI, confidence interval.

*P*-values were determined using logistic regression models. ^*^ *P*<0.05, ^**^ *P*<0.01.

## Discussion

In this study, it was first found that the level of endometrial CD138^+^ cells during the proliferative phase was much higher than that in the mid-luteal phase among the same patients. It was further demonstrated that the presence of ≥3 CD138^+^ plasma cell-positive lesions in the proliferative phase was associated with poor reproductive outcomes in infertile IVF-ET patients compared with the <3 positive lesions group.

Our data demonstrated that CE is a key risk factor for adverse pregnancy outcomes in infertile patients. Therefore, we have shown that endometrial factors are very important for pregnancy outcomes (21, 22). According to the above data, CE patients requiring treatment were defined by whether ≥3 CD138^+^ plasma cell-positive lesions were present in the endometrial stroma during the proliferative phase. The incidence of CE in infertile women is 5.3% according to the CE diagnostic criteria during the luteal phase (4), while it is 14.78% when following the above diagnostic criteria during the proliferative phase. This observation indicated that the incidence of CE changed during the proliferative and mid-luteal phases.

In this study, endometrial tissues of the same patients were taken in the proliferative and mid-luteal phases within 6 months without administration of antibiotics. It was found that CD138^+^ cells during the proliferative phase were significantly higher in number than during the mid-luteal phase. The result concurs with previous reports that the count of CD138^+^ cells can vary in the proliferative and mid-luteal stages of the menstrual period. Inki et al. found that normal endometria in the mid-luteal phase showed negative or few CD138 staining (16). Lai et al. (20) stated that the expression of CD138 in the endometrium stroma was downregulated after ovulation and remained at a low level in the secretory phase. Research by Ryan et al. showed that the number of CD138^+^ cells during the luteal phase decreased in comparison with that in the proliferative phase (19). These results indicate that a certain number of CD138^+^ cells are present in the normal physiological state of the uterine stroma during the proliferative phase, which does not indicate a higher degree of inflammation than in the mid-luteal phase.

The extent to which CE requires clinical treatment is unclear because CE patients usually have no or weak clinical symptoms. In this study, the criteria for clinically treatable CE were defined according to the pregnancy outcomes of infertile patients. Specifically, the correlation between the amount of CD138^+^ cells in the endometrium and the pregnancy outcomes of infertility patients was analyzed to establish the diagnostic criteria for clinically treatable CE in the proliferative phase. One HPF with ≥5 CD138^+^ cells was defined as one positive lesion. It was discovered that ≥3 endometrial lesions with CD138^+^ cells resulted in a significant decrease in live birth, clinical pregnancy, and ongoing pregnancy rates in the proliferative phase compared with patients with <3 positive lesions. However, previous research conducted during the mid-luteal phase indicated that β-hCG positivity, clinical pregnancy, and live birth rates exhibited a significant reduction in women with one lesion with no less than five CD138^+^ cells per HPF (4). These data indicated that more CD138^+^ cells are required during the proliferative phase to impact the pregnancy outcomes of infertile women compared with samples in the mid-luteal phase. Hence, different diagnostic criteria for CE should be established according to the phase of endometrial sampling.

The difference in CD138^+^ cells between the proliferative and mid-luteal phases can be attributed to several factors, including hormonal influence, endometrial microbiota, and sampling depth. First, the cell number and function of the maternal immune system are controlled by hormones, and low endometrial B cells exhibit fluctuations dependent on the menstrual period (23). While progesterone essentially exerts immunosuppressive actions, the immune effects of estrogens are concentration- and context-dependent (24). It is known that estrogens and progesterone can modulate the activation of B cells (25, 26) and the generation of plasma cells (27). Estrogens have been observed to promote the proliferation of B cells, which may result in a higher count of CD138^+^ plasma cells during the proliferative phase. Second, it is reported that the uterus has a resident microbiota including bacteria, viruses, archaea, and fungi (28, 29). Interestingly, the abundance of bacteria, viruses, and archaea shows significant differences between the mid-secretory and proliferative phases, which supports the hypothesis of dependence on the microbiota cycle (29). Therefore, it was speculated that the transformation of microbiota during the menstrual period might affect the number of plasma cells. Third, endometrial thickness in the early proliferative phase is only 5-7 mm but grows to an average of 12-13 mm in the mid-luteal phase, which results in different endometrial sampling depths in the two phases. The proliferative phase has a thinner functional layer than the secretory phase. Thus, biopsies performed during the proliferative phase could obtain more basal layer tissues and plasma cells.

This study has several strengths. First, the number of CD138^+^ cells in the endometrium during the proliferative and mid-luteal phases without any antibiotic treatment among the same patients was compared for the first time. Second, the minimum number of endometrial CD138^+^ cells collected during the proliferative phase to identify treatable CE was determined according to infertile patients’ pregnancy outcomes without being treated with antibiotics. Based on this standard, the pregnancy outcomes of infertile patients could return to the same level as that of non-treatable CE after treatment (data not shown), which proves that the standard has certain clinical applicability. Third, it was first proposed that the number of positive lesions can be used to determine whether CE requires treatment.

Some limitations can be found in this study. First, different sampling methods of endometria in the proliferative and mid-luteal phases may lead to differences in tissue acquisition such as sampling depth or location. Second, this is a single-center and retrospective control study. The study subjects’ clinical and demographic data are not complete. Third, the clinical features of the uteruses observed under hysteroscopy were not recorded completely. The association between the uterine symptoms such as congestion, polyps, and, edema, and the number of CD138 cells could not be obtained. Therefore, some missing confounding factors may exert an impact on the research conclusions, which need to be further verified by carefully designed prospective research. Third, the diagnosis of CE was not combined with IHC and hysteroscopic features in this study because the clinical features under hysteroscopy were not specific and obvious.

## Conclusions

In conclusion, the findings of this study revealed that the number of endometrial CD138^+^ cells during the proliferative phase was much higher than during the mid-luteal phase. Therefore, a higher number of CD138^+^ cells were required during the proliferative phase for treatable CE. Different diagnostic criteria for CE should be created for the proliferative and mid-luteal phases.

## Data Availability

The original contributions presented in the study are included in the article/**Supplementary Material**. Further inquiries can be directed to the corresponding author.

## Supplementary material

Supplementary Figure 1 Pregnancy outcomes among patients in different groups. Based on the distribution of CD138^+^cells in the stroma, patients were grouped the infertile patients into three groups according to the number of positive lesions: no lesion, 1-2 positive lesions, and ≥3 positive lesions. The live birth, clinical pregnancy and biochemical pregnancy rates were determined.
