# Supplementary figures and images for: The effect of the number of endometrial CD138+ cells on the pregnancy outcomes of infertile patients in the proliferative phase
Source: Front Endocrinol (Lausanne). 2025 Jan 22;15:1437781. doi: 10.3389/fendo.2024.1437781 (PMC11794120; doi:10.3389/fendo.2024.1437781)

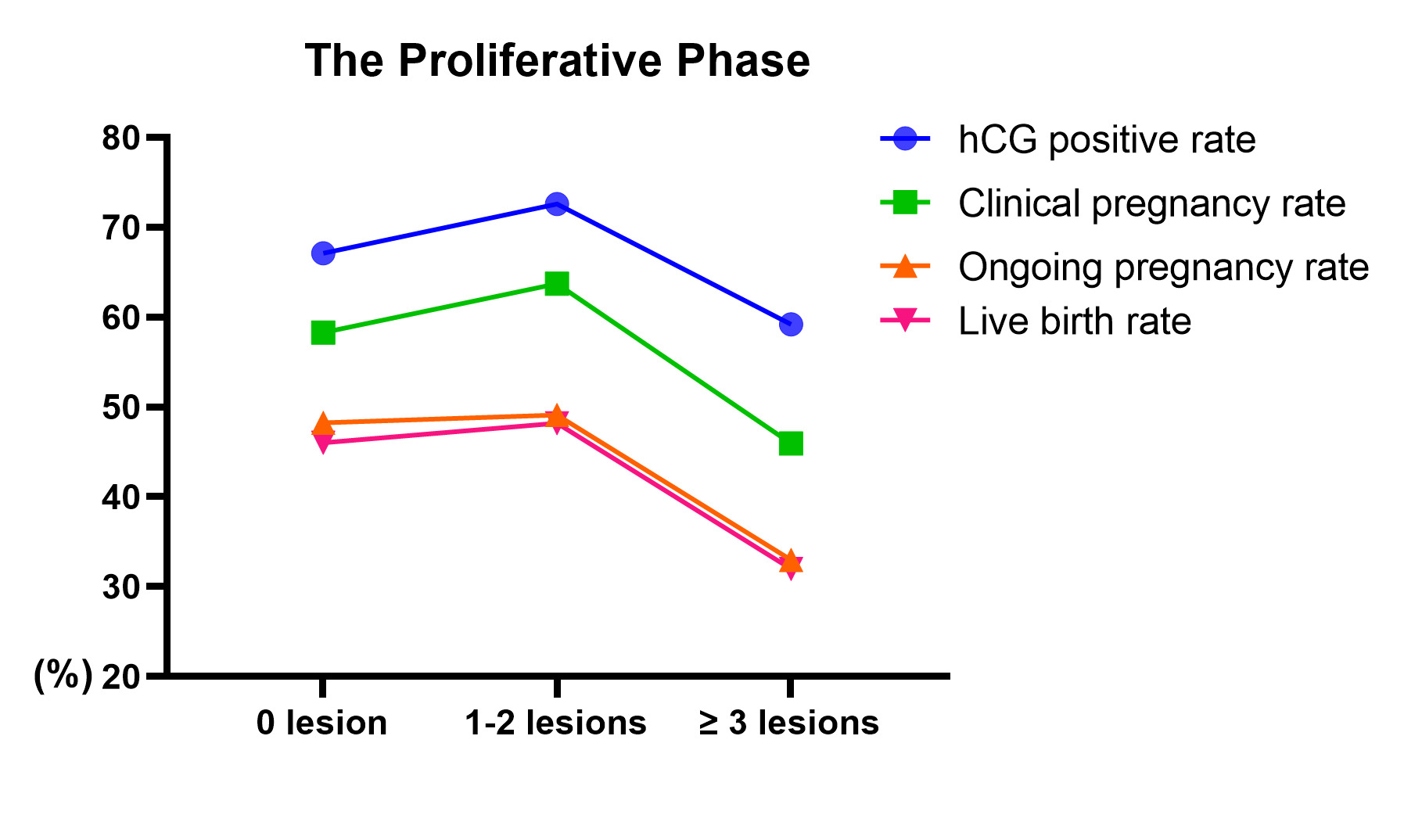

Supplement: Supplementary Figure 1 — Pregnancy outcomes among patients in different groups. Based on the distribution of CD138+cells in the stroma, patients were grouped the infertile patients into three groups according to the number of positive lesions: no lesion, 1-2 positive lesions, and ≥3 positive lesions. The live birth, clinical pregnancy and biochemical pregnancy rates were determined. [file Image1.jpg]
