# Supplementary material for: The effect of the number of endometrial CD138+ cells on the pregnancy outcomes of infertile patients in the proliferative phase
Source: Front Endocrinol (Lausanne). 2025 Jan 22;15:1437781. doi: 10.3389/fendo.2024.1437781 (PMC11794120; doi:10.3389/fendo.2024.1437781)
Supplement: Supplementary file 2 [file DataSheet1.docx]

**Supplementary Table 1 The comparison of pregnancy outcomes of <3 positive lesion and ≥3 positive lesion group**

| **Outcomes** | **<3 positive lesion**  **(n=566)** | **≥3 positive lesion (n=98)** | ***P* value** |
| --- | --- | --- | --- |
| **Live birth rate (%)** | 46.44% (261/562) | 31.96% (31/97) | .008^**^ |
| Biochemical pregnancy rate (%) | 68.20% (386/566) | 59.18% (58/98) | .080 |
| Clinical pregnancy rate (%) | 59.36% (336/566) | 45.92% (45/98) | .013^*^ |
| Ongoing pregnancy rate (%) | 48.40% (272/562) | 32.99% (32/97) | .005^**^ |

Values were presented as column percentage (%). *P* values were determined using Pearson chi-square tests. ^*^*P*＜0.05, ^**^ *P*＜0.01.
