# Supplementary material for: The effect of the number of endometrial CD138+ cells on the pregnancy outcomes of infertile patients in the proliferative phase
Source: Front Endocrinol (Lausanne). 2025 Jan 22;15:1437781. doi: 10.3389/fendo.2024.1437781 (PMC11794120; doi:10.3389/fendo.2024.1437781)
Supplement: Supplementary file 3 [file DataSheet2.docx]

**Supplementary Table 2 Frequency table of positive lesion**

| **Number of positive lesions** | **Frequency** | **Cumulative Frequency** | **Percentage (%)** | **Cumulative percentage (%)** |
| --- | --- | --- | --- | --- |
| **0** | 453 | 453 | 68.2 | 68.2 |
| **1** | 83 | 536 | 12.5 | 80.7 |
| **2** | 30 | 566 | 4.5 | 85.2 |
| **3** | 19 | 585 | 2.9 | 88.1 |
| **4** | 19 | 604 | 2.9 | 91.0 |
| **5** | 11 | 615 | 1.7 | 92.6 |
| **6** | 5 | 620 | 0.8 | 93.4 |
| **7** | 4 | 624 | 0.6 | 94.0 |
| **8** | 7 | 631 | 1.1 | 95.0 |
| **9** | 6 | 637 | 0.9 | 95.9 |
| **10** | 5 | 642 | 0.8 | 96.7 |
| **11** | 4 | 646 | 0.6 | 97.3 |
| **13** | 1 | 647 | 0.2 | 97.4 |
| **14** | 1 | 648 | 0.2 | 97.6 |
| **15** | 1 | 649 | 0.2 | 97.7 |
| **16** | 4 | 653 | 0.6 | 98.3 |
| **19** | 1 | 654 | 0.2 | 98.5 |
| **27** | 2 | 656 | 0.3 | 98.8 |
| **28** | 1 | 657 | 0.2 | 98.9 |
| **30** | 7 | 664 | 1.1 | 100.0 |
